# The role of continuity of care in high-risk pregnant women in Indonesia

**DOI:** 10.18332/ejm/195831

**Published:** 2025-01-03

**Authors:** Siti Mar’atus Sholikah, Fitria Nurwulansari, Elfira Nurul Aini, Slamet Wardoyo, Jessica Juan Pramudita

**Affiliations:** 1 Department of Midwifery, Poltekkes Kemenkes Surabaya, Surabaya, Indonesia; 2 Department of Environmental Health, Poltekkes Kemenkes Surabaya, Surabaya, Indonesia; 3 Department of Blood Bank Technology, Poltekkes Kemenkes Semarang, Semarang, Indonesia

**Keywords:** continuity of care, family independence, health assistance, high-risk pregnant women, prevention of pregnancy complications

## Abstract

**INTRODUCTION:**

High-risk pregnancies require special attention in maternal and child health services, given the high potential for complications that can affect maternal and fetal health. The continuity of care (COC) approach is expected to increase family independence and prevent pregnancy complications. This study aims to analyze the effectiveness of COC in improving the family independence of high-risk pregnant women in preventing pregnancy complications.

**METHODS:**

This study used a quasi-experimental design with a pretest-posttest approach without a control group, involving 134 high-risk pregnant women, in the target area at the Wonoayu Community Health Centre, Sidoarjo, Indonesia from February to May 2024, who were selected through purposive sampling. Data were collected through structured questionnaires before and after the COC intervention, which included assessing knowledge and family roles in supporting pregnant women's health. The COC mentoring program was implemented for three months, with a focus on family education and involvement in maternal healthcare. A validated questionnaire measured family self-reliance before and after the intervention.

**RESULTS:**

The study showed a significant increase in family self-reliance, with a p<0.001 in all aspects measured, including fulfilment of physiological and psychological needs, preparation for labor, the postpartum period, and preparation after the baby is born. This increase suggests that the COC intervention is effective in empowering families to support high-risk pregnant women. Before the intervention, the mean score for physiological and psychological needs fulfilment was 17.45, which increased to 36.42 after the intervention (p<0.001). In addition, labor preparation also showed a significant increase from 11.40 to 24.38, as well as postpartum preparation from 13.00 to 28.79, and preparation after the baby is born from 13.25 to 28.75 (all p<0.001).

**CONCLUSIONS:**

The consistent improvement in all measured aspects, indicated that the COC intervention not only improved families' knowledge and skills, but also contributed to their preparedness in supporting pregnant women during and after pregnancy.

## INTRODUCTION

High-risk pregnancy is a condition that requires special attention in maternal and child health services, due to the high potential for complications that can affect the health of the mother and fetus. Various factors such as health history, socio-economic conditions, and limited access to comprehensive health services often worsen the condition of high-risk pregnant women^1,2^. To meet this challenge, the continuity of care (COC) approach is crucial in ensuring continuity of monitoring and effective interventions. COC is a concept that emphasizes the importance of continuity in the delivery of health services from pregnancy to postpartum, with the aim of increasing family independence in preventing pregnancy complications^3,4^.

One of the main problems in the healthcare of high-risk pregnant women is the lack of continuity of care received by women during pregnancy and postpartum. Studies show that this lack of continuity of care is often due to differences in health providers, limited access to health facilities, and lack of knowledge among mothers and families about measures that can be taken to prevent pregnancy complications^5,6^. This condition is exacerbated by the limited role of families in supporting pregnant women undergoing high-risk pregnancies. In fact, the role of the family is very important in ensuring that pregnant women get proper care and comply with medical advice^7,8^.

Recent studies have shown that implementing COC in high-risk pregnant women can lead to better pregnancy outcomes, including reduced rates of complications such as preeclampsia, preterm birth, and other labor complications^9,10^. COC involves multidisciplinary collaboration between midwives, doctors, and other health professionals who work together to provide coordinated and continuous care. A study by Hardiningsih et al.^11^ found that pregnant women who received services using the COC approach showed increased independence in making health-related decisions, including in terms of nutritional intake, physical activity, and appropriate use of health services. In addition, this approach also helps in early detection of complications, so that medical interventions can be carried out more quickly and effectively.

While the benefits of COC have been recognized in various studies, there are several challenges that require further attention. One of the main challenges is the suboptimal implementation of COC in high-risk pregnant women, especially in developing countries. Limited human resources and health infrastructure are often the main barriers to equal access to COC services, especially in remote areas^6,12^. In addition, most studies on COC emphasize clinical outcomes, while other important aspects such as family self-reliance in preventing complications during pregnancy are less explored. Family empowerment through education and support is a crucial component in the successful implementation of COC^13,14^. Therefore, further research is needed to identify the role of COC in improving family self-reliance, particularly in resource-limited countries, to ensure the effectiveness of preventing pregnancy complications in high-risk pregnant women. This study aims to evaluate the role of COC in improving family self-reliance in supporting the health of high-risk pregnant women.

## METHODS

### Research design and setting

This study used a quantitative approach with a quasi-experimental design. The design applied was a non-randomized pretest-posttest without control group design, which allows measuring changes in family self-reliance in the prevention of pregnancy complications in high-risk pregnant women before and after the continuity of care (COC) intervention without comparing with a control group.

The study was conducted at Puskesmas Wonoayu, Sidoarjo, Indonesia, which is a primary healthcare facility serving the community in its working area. This health center was chosen because it had a sufficient number of high-risk pregnant women to meet the study inclusion and exclusion criteria. The participant recruitment process took place from February to May 2024, and data collection was conducted in stages during this period, with a one-month post-intervention follow-up to measure changes in family independence.

Participants in this study were pregnant women with high-risk criteria who met the inclusion requirements, such as maternal age, relevant medical history, and multiple pregnancies.

### Participants

This study involved 134 high-risk pregnant women from the working area of Puskesmas Wonoayu, Sidoarjo, Indonesia. The sampling method used was purposive sampling, to ensure that the participants involved matched the required high-risk characteristics. Participants were divided by trimester of pregnancy to ensure representative distribution. Inclusion criteria were: pregnant women with first to third trimester gestational age; having a high-risk pregnancy (having hypertension, gestational diabetes); previous history of pregnancy complications; and being able to communicate in Indonesian. Additional criteria included willingness to involve family members in the COC intervention, as the family plays an important role in the complication prevention strategies implemented.

### Ethics

This study was approved by the Health Research Ethics Committee of Poltekkes Kemenkes Surabaya with registration number EA/2689/KEPK-Poltekkes_Sby/V/2024 dated 13 November 2023. Before the study began, all respondents provided informed consent, after explanation of the purpose of the study, intervention procedures, and their rights as participants. Respondents had the right to withdraw from the study at any time without any consequences. The data collected from the respondents will be kept confidential and only used for research purposes.

### Study variables

The independent variable in this study was the COC intervention, which involves a series of ongoing health monitoring services for high-risk pregnant women. The dependent variable was the level of family role in preventing pregnancy complications, which was measured through the family’s ability to recognize risk signs, take preventive action, and proactively accompany pregnant women across the following domains: fulfilment of physiological and psychological needs; labor preparation; postpartum preparation; and preparation after the baby is born.

This level of independence was assessed before and after the intervention to observe changes as a result of COC. Other factors assessed included maternal age, gestational age, socioeconomic status, medical history, and family support. Maternal age and gestational age may influence readiness to receive information and take preventive measures, while socioeconomic status relates to access to health resources and family financial preparedness. Health history, which includes the presence of chronic diseases or previous pregnancy complications, was also considered as it may increase the need for more intensive health support. Unequal family support in accompanying pregnant women was also an important variable, as family independence is highly dependent on the involvement of family members.

### Intervention

The intervention in this study was a COC program designed to improve family self-reliance in the prevention of pregnancy complications in high-risk pregnant women. The intervention focuses on providing health education, regular monitoring, and active family involvement in the care of pregnant women during pregnancy, to reduce potential complications that may arise. The intervention was delivered in the form of face-to-face meetings and counselling sessions, where pregnant women and family members were provided with information on pregnancy danger signs, prevention measures, and the importance of ongoing health care.

Participants in the COC intervention received routine pregnancy monitoring at Puskesmas Wonoayu, including health checks by midwives and other health workers. They were also provided with educational materials on danger signs during pregnancy, ways to prevent complications, and the importance of family support in maintaining the health of pregnant women. Each participant received counselling sessions conducted by an experienced midwife, who was also responsible for providing training on family health monitoring. During the intervention, families were taught how to monitor the condition of pregnant women, such as blood pressure and watch for signs of complications that should be reported immediately.

To avoid any bias in the delivery of the intervention, all participants receiving COC were given similar treatment, with no other treatments or exposures that could affect the results. All participants in the intervention group followed the same program, with interventions delivered by a trained and standardized team to ensure quality delivery of information and monitoring of maternal health. To ensure consistent intervention delivery and reduce inter-operator variability, training and standardization of procedures were conducted for all health workers involved, especially the midwives who conducted the education and monitoring sessions. There were five midwives involved in data collection, and they were trained to use uniform instruments and follow the same protocol during data collection and intervention delivery. With an uncontrolled pretest-posttest design, the intervention was closely monitored to ensure that changes in family self-reliance could be attributed to COC delivery and not influenced by other uncontrolled variables.

### Data sources and measurements

Data sources in this study were obtained through primary data collection from high-risk pregnant women who participated in the COC program. Data were collected using a structured questionnaire completed by the participant’s family before and after the COC intervention. Data measurement was conducted using a structured questionnaire designed to assess the level of family self-reliance in preventing pregnancy complications, as well as to collect information related to the demographics, clinical conditions, and maternity history of pregnant women. Data were collected through direct interviews and questionnaires completed by participants (both pregnant women and family members involved in mentoring).

The questionnaire used consisted of two main sections: the first section collected demographic data (e.g. age, education level, employment status, and socioeconomic status), while the second section measured the level of family self-reliance in supporting pregnant women. Family self-reliance was measured through a series of closed-ended questions, using a Likert scale (1–5) to assess the extent to which the family could recognize danger signs, take preventive actions, and provide support to pregnant women.

To measure the level of family self-reliance, this questionnaire was developed by the research team by considering relevant literature and previous research results. The questionnaire was pretested on a small group of pregnant women in another health center before being used in the main study to ensure clarity of questions and to identify features that required modification. Preliminary testing yielded a Cronbach’s alpha coefficient of 0.85, indicating a good level of reliability for use in this population. Validity testing was also conducted by involving experts in the field of maternal and family health to ensure the content of the questionnaire was relevant to the research objectives. The questionnaire also included open-ended questions regarding challenges faced by the family in supporting the pregnant woman and preventive measures taken.

All data were collected at two points in time: before and after the COC intervention, to assess changes in family self-reliance. The data were collected by five trained midwives, who were trained in interview techniques and questionnaire completion to ensure consistency in data collection and minimize inter-operator bias.

### Statistical analysis

The data obtained were analyzed using descriptive statistical tests to see the frequency distribution and sample characteristics. A comparative test using independent t-test was conducted to test the difference between the control and intervention groups. To test the difference between pretest and posttest in the intervention group, paired t-test was used. The statistical package XXX was used to perform the statistical analysis.

## RESULTS

Table 1 provides an overview of the characteristics of the participants of this study, which consisted of 134 pregnant women categorized as high risk. The majority of respondents were in the first trimester of pregnancy (58.2%), followed by the second (29.0%) and third (11.9%) trimesters. In terms of age, most participants (73.9%) were aged 20–35 years, which is the ideal age range for pregnancy, while only 11.19% were aged <20 years, and 14.93% were aged >35 years. The time between marriage and first pregnancy for 90% of the participants was <4 years, indicating their readiness to start a family. Pregnancy spacing showed that 47.01% of the participants had a pregnancy spacing of ≤2 years, and 97% had <4 children, indicating that many of them were relatively new expectant mothers. Lastly, 73.9% of participants had a normal pregnancy history, indicating previous positive experiences in pregnancy.

**Table 1:** Characteristics of the high-risk pregnant women that participated from the Wonoayu Community Health Centre, Sidoarjo, Indonesia, February – May 2024 (N=134)

*Characteristics*	*n*	*%*
**Pregnancy age**		
Trimester 1	78	58.21
Trimester 2	40	29.90
Trimester 3	16	11.94
**Age** (years)		
≤20	15	11.19
21–35	99	73.88
>35	20	14.93
**Time from marriage to first pregnancy** (years)		
<4	120	90.00
≥4	14	10.00
**Body height** (cm)		
<145	53	39.55
≥145	81	60.45
**Body weight** (kg)		
<45	28	20.90
≥45	106	79.10
**Pregnancy spacing** (years)		
≤2	63	47.01
3–9	67	50.00
≥10	4	02.99
**Number of children**		
<4	130	97.01
≥4	4	02.99
**Pregnancy history**		
Normal	99	73.88
Not normal	35	26.12

Table 2 shows significant results regarding the effectiveness of COC-based interventions on family independence in preventing pregnancy complications. Before the intervention, the mean score for physiological and psychological needs fulfilment was 17.45, which increased to 36.42 after the intervention (p<0.001). In addition, labor preparation also showed a significant increase from 11.40 to 24.38, as well as postpartum preparation from 13.00 to 28.79, and preparation after the baby is born from 13.25 to 28.75 (all p<0.001).

**Table 2:** Changes in the scores across four domains reflecting the role of family members of high-risk pregnant women before and after a COC-based assistance program among high-risk pregnant women from the Wonoayu Community Health Centre, Sidoarjo, Indonesia, February – May 2024 (N=134)

*Domains*	*n Before*	*n After*	*Mean Before*	*Mean After*	*SD Before*	*SD After*	*p*
Fulfilment of physiological and psychological needs	134	134	17.45	36.42	2.63	5.51	0.001
Labor preparation	134	134	11.40	24.38	2.41	3.51	0.001
Postpartum preparation	134	134	13.00	28.79	2.19	4.07	0.001
Preparation after the baby is born	134	134	13.25	28.75	2.14	4.21	0.001

## DISCUSSION

This study aimed to assess the role of COC in high-risk pregnant women in increasing family independence in preventing pregnancy complications. Based on the results obtained, this study shows that the COC intervention has a significant effect in increasing family knowledge and skills in supporting the health of pregnant women, especially in preventing pregnancy complications. Family independence in recognizing danger signs, taking preventive actions, and supporting the care process of pregnant women, increased significantly after the COC intervention. This reflects the importance of family involvement in the health management of pregnant women, especially for those in high-risk groups.

The success of the COC program in this study highlights the importance of integrated and sustainable health service provision, which involves not only pregnant women, but also families as part of the support system. This study shows that providing interventions that actively involve families can be an effective strategy in reducing the risk of pregnancy complications and increasing family understanding of the importance of early prevention.

Based on the results of this study, it was found that the implementation of COC in high-risk pregnant women can increase the understanding and independence of families in facing various pregnancy challenges. The COC approach allows mothers and their families to receive clear and targeted information about the pregnancy risks they face, so that they can take appropriate preventive measures^15^. In this case, COC acts not only as an intervention but also as an educational tool that can increase family independence in maintaining the health of pregnant women and preventing complications.

Previous research has indicated that pregnant women who received COC were more confident in managing their pregnancy, especially in making decisions related to a healthy lifestyle, such as diet, physical activity, and adherence to medical advice^13^. A study by Bradford et al.^6^ showed that COC is associated with improved maternal health outcomes, including reduced rates of preterm birth and other pregnancy complications.

The findings in this study are also supported by other studies that emphasize the importance of COC in maintaining the health of high-risk pregnant women. For example, a study found that mothers who received COC, tended to be better able to cope with health risks compared to mothers who received fragmented care^16^. In addition, other studies have also showed that COC plays a role in increasing pregnant women’s satisfaction with the health services they receive^17^. This satisfaction arises due to the close relationship between the mother and the healthcare provider, which can create a sense of security and comfort for the mother in undergoing a high-risk pregnancy. This relationship also contributes to increased family independence, as the family can be directly involved in the decision-making process based on accurate information from the provider.

However, despite the overwhelming evidence showing the benefits of COC, several studies have found that COC implementation still faces challenges. Bradford et al.^6^ noted that in some developing countries, limited access to sustainable health services is often a major obstacle to COC implementation, especially in remote areas. This can lead to a break in the chain of care, which in turn negatively affects the health of pregnant women^6^.

Improved family self-reliance through COC in this study was seen in various aspects. Families are not only more informed about their pregnancy condition, but are also more proactive in ensuring that pregnant women receive appropriate care^14^. This shows that education provided through COC can empower families to prevent complications that may arise during pregnancy, such as preeclampsia and preterm labour^13,14^. This family independence is in line with the concept of empowerment emphasized in maternal health literature which has noted that family empowerment through continuing education can increase family involvement in the care of pregnant women, leading to improved overall pregnancy outcomes. With a better understanding, the family can support the mother in leading a healthy lifestyle, adhering to the pregnancy control schedule, and taking prompt action if signs of complications arise^14^.

### Limitations

Although this study shows significant benefits of implementing COC, there are some limitations that should be noted. Firstly, this study only included samples from urban areas, so the results may not be representative of high-risk pregnant women in rural or remote areas, where limited health infrastructure is often a major obstacle to optimal COC implementation. The limited number of health workers and medical facilities in these areas may affect the quality and continuity of care^18^. Secondly, the long-term impact of COC on maternal and infant health has not been fully explored in this study. Further studies are needed to evaluate how the implementation of COC may affect the health of high-risk mothers postpartum, including support for physical and mental recovery, which was also recognized as important by the study^13^. Another limitation is the lack of explanation of the interaction between healthcare providers and families, which is crucial in determining the extent to which information and support provided is implemented by families^19,20^. Therefore, further research should include a deeper analysis of the relationship dynamics between service providers and families in the context of COC implementation.

## CONCLUSIONS

Overall, this study shows that COC plays an important role in improving family self-reliance in dealing with high-risk pregnancies, while contributing to the prevention of pregnancy complications. Although there are some limitations to its implementation, the benefits of the continuity of care approach are clear. However, further research is needed to explore the long-term impact of COC and to address limitations in its application, particularly in areas with limited access to health services.

## Data Availability

The data supporting this research are available from the authors on reasonable request.
