# Erratum: The role of continuity of care in high-risk pregnant women in Indonesia

**DOI:** 10.18332/ejm/ejm/200122

**Published:** 2025-01-17

**Authors:** European Publishing Production Team

**Affiliations:** 1 European Publishing Production Team, Heraklion, Greece

**Keywords:** continuity of care, family independence, health assistance, high-risk pregnant women, prevention of pregnancy complications

**Erratum on: The role of continuity of care in high-risk pregnant women in Indonesia**

**By Siti Mar’atus Sholikah, Firia Nurwulansari, Elfira Nurul Aini, Slamet Wardoyo, Jessica Juan Pramudita**

**European Journal of Midwifery, Volume 9, Issue January, Pages 1-6**

**Publish date: 3 January 2025**

**DOI:** https://doi.org/10.18332/ejm/195831

In the original article, the article number was mistakenly given as follows:

Eur J Midwifery 2025;9(January):78

The article number in the original manuscript is now corrected:

Eur J Midwifery 2025;9(January):2

The mentioned change is corrected also online.

